# Supplementary material for: Insight into the bioactivity and action mode of betulin, a candidate aphicide from plant metabolite, against aphids
Source: eLife. 2025 Nov 3;14:RP107598. doi: 10.7554/eLife.107598 (PMC12582564; doi:10.7554/eLife.107598)
Supplement: Figure 1—source data 1. [file elife-107598-fig1-data1.docx]

**Figure 1—Source Data 1.** LC_50_ values of betulin and pymetrozine against *M. persicae* at 48 h, corresponding to Figure 1, panel D and F.

| **Compounds** | **Regression equation** | **LC_50_**  **(mg⋅mL^−1^)** | **95% Confidence**  **Interval (mg⋅mL^−1^)** | ***r^2^*** |
| --- | --- | --- | --- | --- |
| betulin | Y=6.9323+2.4621X | 0.1641 | 0.1635-0.1648 | 0.9235 |
| pymetrozine | Y=4.9840+0.6195X | 1.0612 | 1.0037-1.1221 | 0.9806 |
