# Supplementary material for: Insight into the bioactivity and action mode of betulin, a candidate aphicide from plant metabolite, against aphids
Source: eLife. 2025 Nov 3;14:RP107598. doi: 10.7554/eLife.107598 (PMC12582564; doi:10.7554/eLife.107598)
Supplement: Figure 3—source data 1. [file elife-107598-fig3-data1.docx]

**Figure 3—Source Data 1.** Complete sequence information for the *GABA_A_ receptor* gene of *M. persicae* corresponding to Figure 3, panel A.

| **Gene** | **Gene ID** | **Coding sequence (bp)** | **Deduced amino acid** | **Molecular weight (kDa)** | **Isoelectric point** |
| --- | --- | --- | --- | --- | --- |
|  |  |  |  |  |  |
|  |  |  |  |  |  |
| MpGABR | 111036118 | 2082 | 693 | 77.20 | 9.85 |
| MpGABRAP | 111041856 | 357 | 118 | 14.08 | 9.63 |
| MpGABRB | 111036117 | 753 | 250 | 28.01 | 7.20 |
