# Supplementary material for: Insight into the bioactivity and action mode of betulin, a candidate aphicide from plant metabolite, against aphids
Source: eLife. 2025 Nov 3;14:RP107598. doi: 10.7554/eLife.107598 (PMC12582564; doi:10.7554/eLife.107598)
Supplement: Figure 3—source data 2. [file elife-107598-fig3-data2.zip › Figure 3—Source Data 2.pdf]

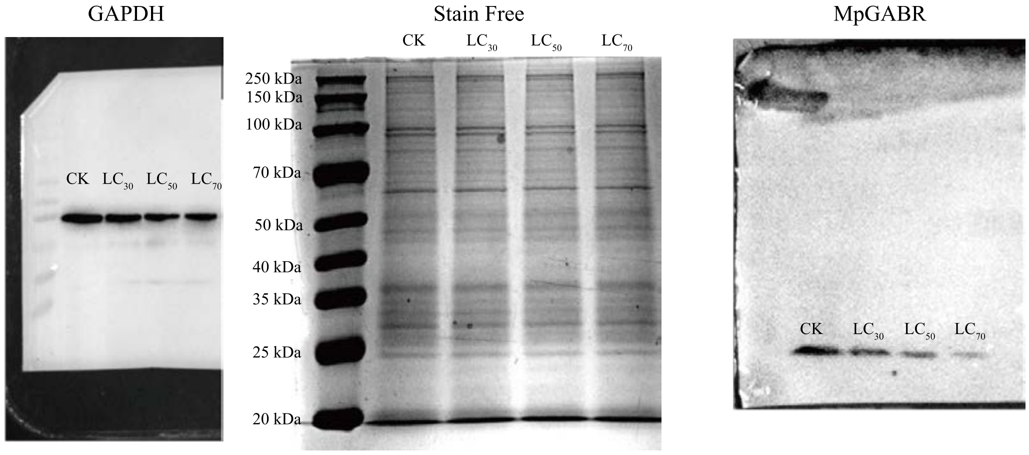

Figure 3—Source Data 2. Original membranes corresponding to Figure 3, panel E. GAPDH was used as a reference protein.
