# Supplementary figures and images for: Insight into the bioactivity and action mode of betulin, a candidate aphicide from plant metabolite, against aphids
Source: eLife. 2025 Nov 3;14:RP107598. doi: 10.7554/eLife.107598 (PMC12582564; doi:10.7554/eLife.107598)

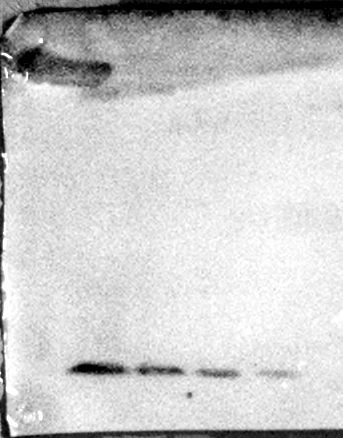

Supplement: Figure 3—source data 3. [file elife-107598-fig3-data3.zip › MpGABR.Tif]

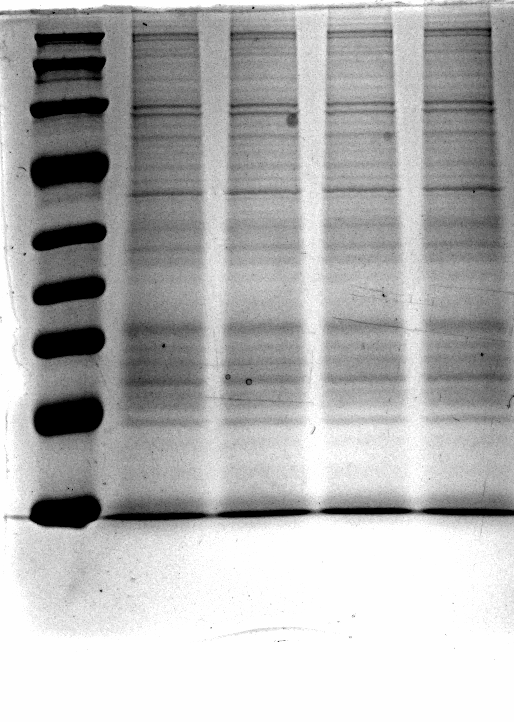

Supplement: Figure 3—source data 3. [file elife-107598-fig3-data3.zip › Stain Free.Tif]

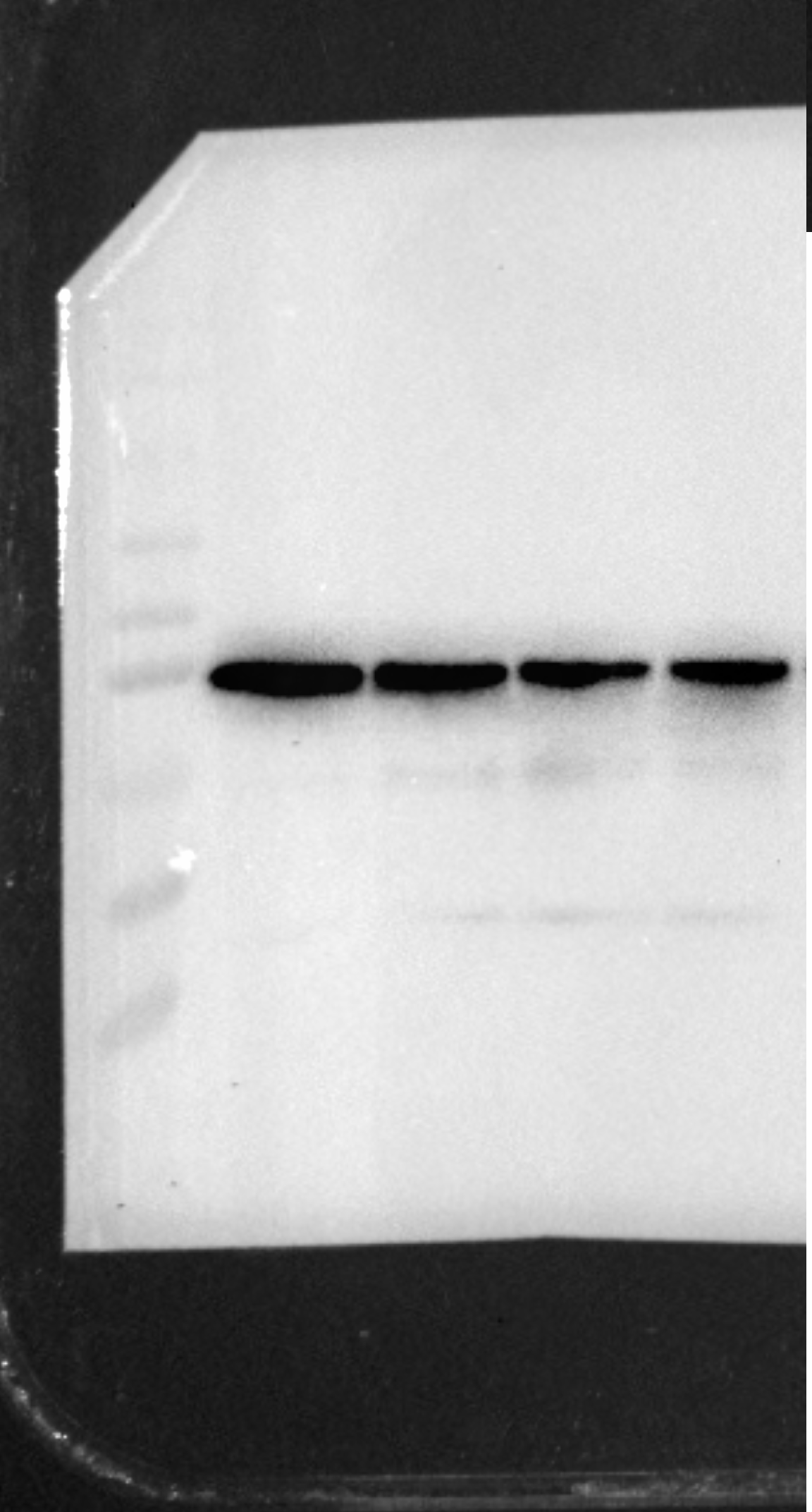

Supplement: Figure 3—source data 3. [file elife-107598-fig3-data3.zip › GAPDH.tif]

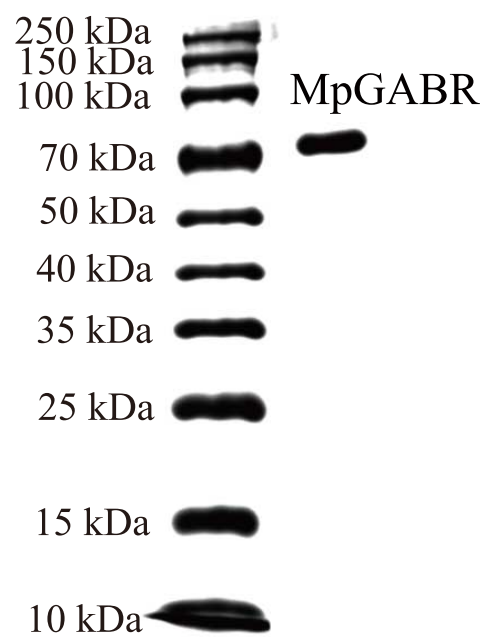

**Figure 6—Source Data 1.** Original gel corresponding to Figure 6, panel A.

Supplement: Figure 6—source data 1. [file elife-107598-fig6-data1.zip › Figure 6—Source Data 1.pdf]

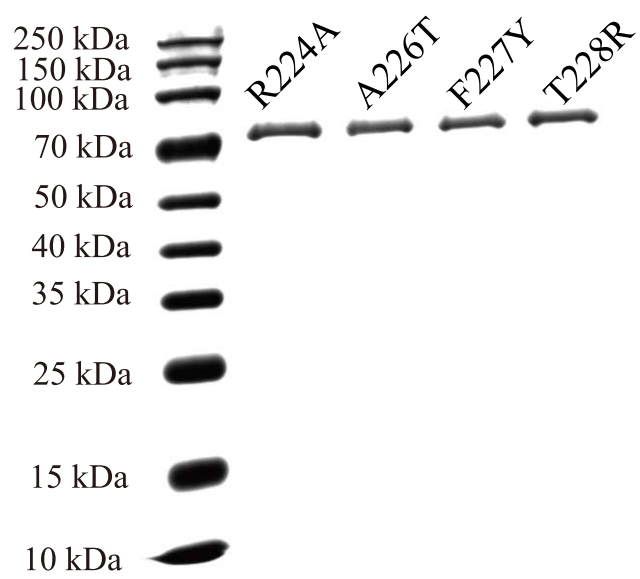

**Figure 7—Source Data 2.** Original gel corresponding to Figure 7, panel C.

Supplement: Figure 7—source data 2. [file elife-107598-fig7-data2.zip › Figure 7—Source Data 2.pdf]

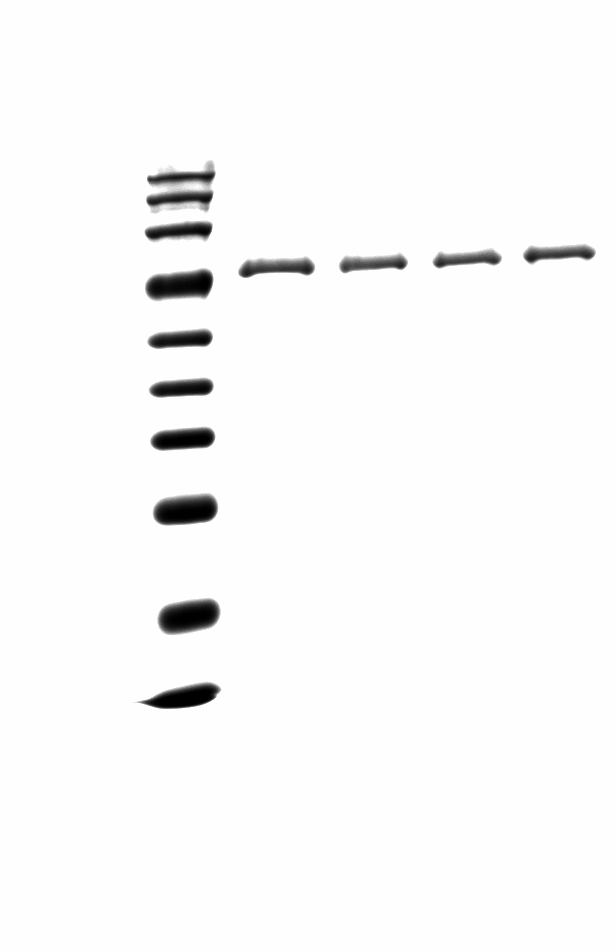

Supplement: Figure 7—source data 3. [file elife-107598-fig7-data3.zip › MpGABR mutations.Tif]

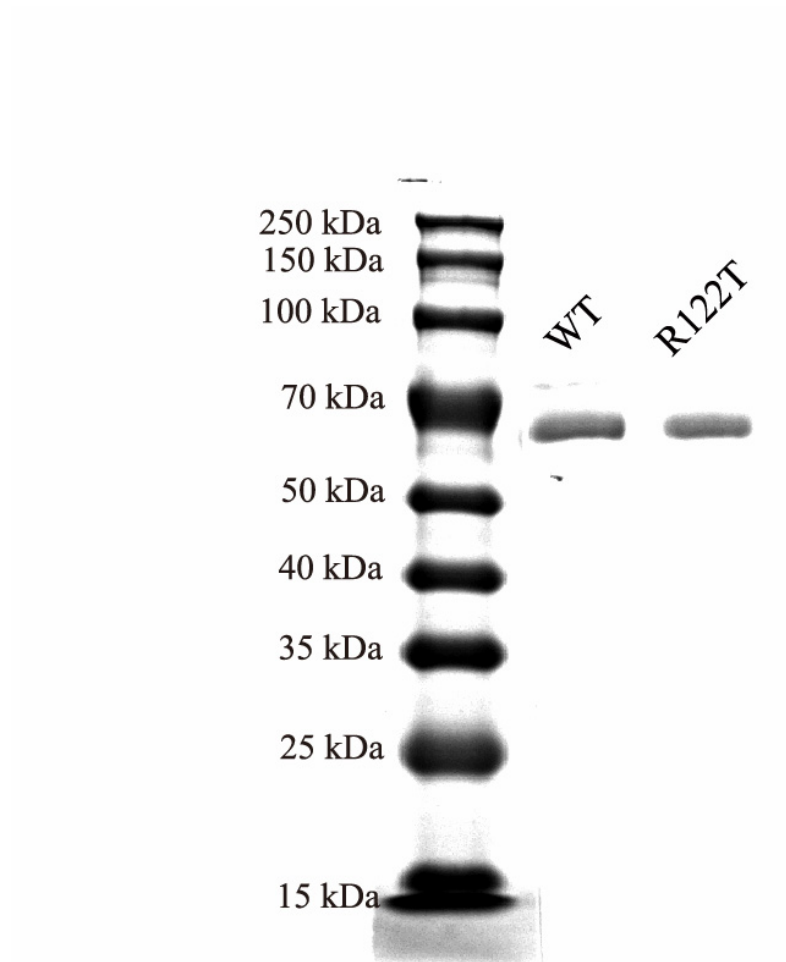

**Figure 8—Source Data 1.** Original gel corresponding to Figure 8, panel A.

Supplement: Figure 8—source data 1. [file elife-107598-fig8-data1.zip › Figure 8—Source Data 1.pdf]

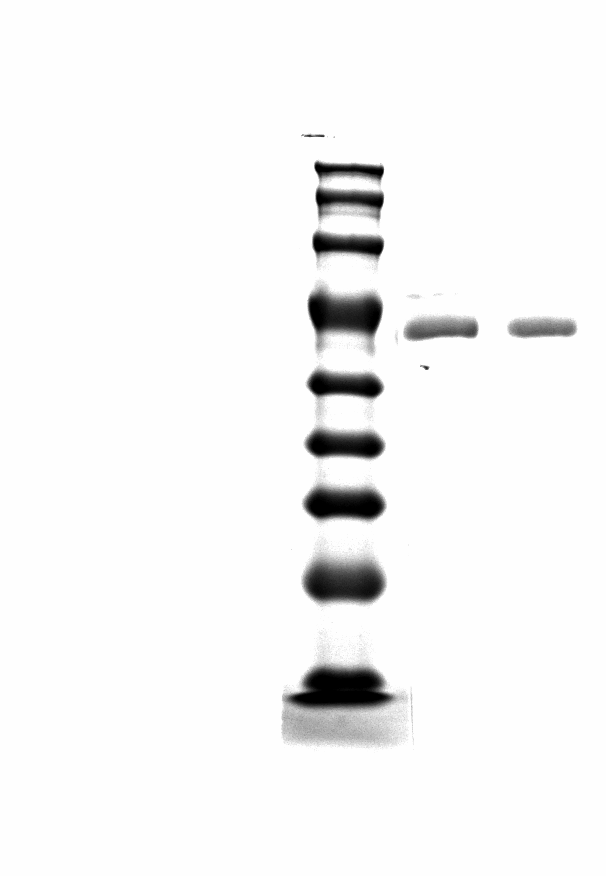

Supplement: Figure 8—source data 2. [file elife-107598-fig8-data2.zip › DmGABR.Tif]
