# Supplementary material for: Insight into the bioactivity and action mode of betulin, a candidate aphicide from plant metabolite, against aphids
Source: eLife. 2025 Nov 3;14:RP107598. doi: 10.7554/eLife.107598 (PMC12582564; doi:10.7554/eLife.107598)
Supplement: Figure 5—source data 1. [file elife-107598-fig5-data1.docx]

**Figure 5—Source Data 1.** Sequences and relevant information for phylogenetic analysis of GABA_A_ receptor

| **Species** | **Accession or GenBank No.** | **Order** |
| --- | --- | --- |
| *Myzus persicae* | XP_022173711.1 | Hemiptera |
| *Metopolophium dirhodum* | XP_060864885.1 | Hemiptera |
| *Acyrthosiphon pisum* | XP_008183008.2 | Hemiptera |
| *Macrosiphum euphorbiae* | CAI6365831.1 | Hemiptera |
| *Rhopalosiphum padi* | XP_060842789.1 | Hemiptera |
| *Melanaphis sacchari* | XP_025199515.1 | Hemiptera |
| *Aphis gossypii* | XP_050054462.1 | Hemiptera |
| *Diuraphis noxia* | XP_015376355.1 | Hemiptera |
| *Sipha flava* | XP_025404819.1 | Hemiptera |
| *Cinara cedri* | VVC42195.1 | Hemiptera |
| *Adelges cooleyi* | XP_050429873.1 | Hemiptera |
| *Laodelphax striatellus* | RZF38914.1 | Hemiptera |
| *Macrosteles quadrilineatus* | XP_054276917.1 | Hemiptera |
| *Planococcus citri* | XP_065213924.1 | Hemiptera |
| *Cyrtorhinus lividipennis* | AHW29556.1 | Hemiptera |
| *Bemisia tabaci* | XP_018901515.2 | Hemiptera |
| *Cimex lectularius* | XP_024083544.1 | Hemiptera |
| *Halyomorpha halys* | XP_014281964.1 | Hemiptera |
| *Anopheles gambiae* | XP_001688775.1 | Diptera |
| *Aedes aegypti* | XP_021696320.1 | Diptera |
| *Sitodiplosis mosellana* | XP_055320038.1 | Diptera |
| *Culicoides brevitarsis* | XP_063702909.1 | Diptera |
| *Lutzomyia longipalpis* | XP_055686441.1 | Diptera |
| *Ceratitis capitata* | XP_004525611.1 | Diptera |
| *Phlebotomus papatasi* | XP_055701411.1 | Diptera |
| *Drosophila persimilis* | XP_026849499.1 | Diptera |
| *Drosophila melanogaster* | AHE41089.1 | Diptera |
| *Drosophila simulans* | AAK00512.1 | Diptera |
| *Plutella xylostella* | NP_001292463.1 | Lepidoptera |
| *Papilio xuthus* | XP_013179056.1 | Lepidoptera |
| *Papilio polytes* | XP_013133342.1 | Lepidoptera |
| *Pieris rapae* | XP_022123609.1 | Lepidoptera |
| *Danaus plexippus* | XP_032527541.1 | Lepidoptera |
| *Amyelois transitella* | XP_013189102.1 | Lepidoptera |
| *Ostrinia furnacalis* | XP_028160229.1 | Lepidoptera |
| *Chilo suppressalis* | ASY91958.1 | Lepidoptera |
| *Trichoplusia ni* | XP_026735745.1 | Lepidoptera |
| *Heliothis virescens* | AAB62572.1 | Lepidoptera |
| *Spodoptera frugiperda* | XP_035445711.1 | Lepidoptera |
| *Bombyx mandarina* | XP_028032124.1 | Lepidoptera |
| *Bombyx mori* | NP_001093294.1 | Lepidoptera |
| *Manduca sexta* | XP_030028507.1 | Lepidoptera |
| *Frankliniella occidentalis* | XP_026285852.1 | Thysanoptera |
| *Thrips palmi* | XP_034255608.1 | Thysanoptera |
| *Neodiprion lecontei* | XP_015509151.1 | Hymenoptera |
| *Athalia rosae* | XP_012264889.1 | Hymenoptera |
| *Cephus cinctus* | XP_015589073.1 | Hymenoptera |
| *Orussus abietinus* | XP_023289674.1 | Hymenoptera |
| *Chelonus insularis* | XP_034938828.1 | Hymenoptera |
| *Microplitis demolitor* | XP_008549804.1 | Hymenoptera |
| *Nasonia vitripennis* | XP_016845786.2 | Hymenoptera |
| *Ceratosolen solmsi marchali* | XP_011495770.1 | Hymenoptera |
| *Camponotus floridanus* | XP_011252265.1 | Hymenoptera |
| *Harpegnathos saltator* | XP_011137565.1 | Hymenoptera |
| *Megachile rotundata* | XP_012143665.1 | Hymenoptera |
| *Osmia lignaria* | XP_034181602.1 | Hymenoptera |
| *Eufriesea mexicana* | XP_017755009.1 | Hymenoptera |
| *Apis dorsata* | XP_006619002.1 | Hymenoptera |
| *Apis mellifera* | XP_006565169.1 | Hymenoptera |
| *Bombus impatiens* | XP_024220796.1 | Hymenoptera |
| *Agrilus planipennis* | XP_018318732.1 | Coleoptera |
| *Photinus pyralis* | XP_031346787.1 | Coleoptera |
| *Nicrophorus vespilloides* | XP_017787270.1 | Coleoptera |
| *Onthophagus taurus* | XP_022907694.1 | Coleoptera |
| *Tribolium castaneum* | NP_001107809.1 | Coleoptera |
| *Tribolium madens* | XP_044271991.1 | Coleoptera |
| *Aethina tumida* | XP_019870942.1 | Coleoptera |
| *Anoplophora glabripennis* | XP_018564077.1 | Coleoptera |
| *Leptinotarsa decemlineata* | XP_023012203.1 | Coleoptera |
| *Oulema oryzae* | BAM66322.1 | Coleoptera |
