# Supplementary material for: Insight into the bioactivity and action mode of betulin, a candidate aphicide from plant metabolite, against aphids
Source: eLife. 2025 Nov 3;14:RP107598. doi: 10.7554/eLife.107598 (PMC12582564; doi:10.7554/eLife.107598)
Supplement: Figure 7—source data 1. [file elife-107598-fig7-data1.docx]

**Figure 7—Source Data 1.** Binding energy and nonbonding interactions between betulin and GABA_A_ receptor, corresponding to Figure 7, panel A.

| **Compound** | **Binding Energy (**kcal⋅mol^–1^**)** | **van der Waals** | **H-Bond** | | **Hydrophobic Interaction** | |
| --- | --- | --- | --- | --- | --- | --- |
|  |  |  | **Amino Acid-**  **Ligand Atom** | **Distance (Å)** | **Amino Acid-Ligand Atom** | **Distance (Å)** |
| betulin | -6.38 | PHE172, GLY174, TYR176 | ALA226 [C…H]  THR228 [C-O.H] | 3.31  2.16 | ARG224 [Alkyl]  ALA226 [Alkyl]  ALA226 [Alkyl]  PHE227 [Pi–Alkyl]  PHE227 [Pi–Alkyl] | 4.29  4.36  5.11  4.16  5.32 |
