# Supplementary material for: Insight into the bioactivity and action mode of betulin, a candidate aphicide from plant metabolite, against aphids
Source: eLife. 2025 Nov 3;14:RP107598. doi: 10.7554/eLife.107598 (PMC12582564; doi:10.7554/eLife.107598)
Supplement: Figure 7—source data 4. [file elife-107598-fig7-data4.docx]

**Figure 7—Source Data 4.** Parameters of dose-response curves from microscale thermophoresis experiments, corresponding to Figure 7, panel D.

| **Compound** | | **Strains** | ***K_d_* (****μM)** | **95% CI ^a^ (μM)** | **HillSlope** | ***r^2^*** |
| --- | --- | --- | --- | --- | --- | --- |
| betulin | WT | 2.24 | 1.860-2.687 | 0.0726 | 0.9864 |  |
|  | R224A | 2.23 | 1.799-2.753 | 0.0827 | 0.9822 |  |
|  | A226T | 2.27 | 1.958-2.628 | 0.0538 | 0.9918 |  |
|  | F227Y | 2.25 | 1.860-2.690 | 0.0823 | 0.9850 |  |
|  | T228R | 5321 | 4599-6152 | 0.0560 | 0.9916 |  |

CI ^a^, Confidence Interval; *R^2^*, coefficient of determination.
