# Supplementary material for: Insight into the bioactivity and action mode of betulin, a candidate aphicide from plant metabolite, against aphids
Source: eLife. 2025 Nov 3;14:RP107598. doi: 10.7554/eLife.107598 (PMC12582564; doi:10.7554/eLife.107598)
Supplement: Figure 8—source data 3. [file elife-107598-fig8-data3.docx]

**Figure 8—Source Data 3.** LD_50_ values of betulin and pymetrozine against *D. melanogaster* at 72 h, corresponding to Figure 8, panel E and F.

| **Compounds** | **Strains** | **Regression**  **equation** | **LD_50_**  **(μg⋅fly^−1^)** | **95% Confidence**  **Interval (μg⋅fly^−1^)** | ***r^2^*** |
| --- | --- | --- | --- | --- | --- |
| betulin | WT | - | > 1000 ^a^ | - | - |
|  | R122T | Y=3.6687+0.9282X | 27.1853 | 25.8872-28.5485 | 0.9944 |
| pymetrozine | WT | Y=5.8681+1.3720X | 0.2330 | 0.2311-0.2348 | 0.9933 |
|  | R122T | Y=5.9388+1.4937X | 0.2352 | 0.2335-0.2369 | 0.9903 |

^a^ Toxicity bioassays were not performed with concentrations exceeding 1000 μg⋅fly^−1^.
