# Supplementary material for: Insight into the bioactivity and action mode of betulin, a candidate aphicide from plant metabolite, against aphids
Source: eLife. 2025 Nov 3;14:RP107598. doi: 10.7554/eLife.107598 (PMC12582564; doi:10.7554/eLife.107598)
Supplement: Supplementary file 1. [file elife-107598-supp1.docx]

**Table S****1.** Primers used for cloning, RNAi, heterologous expression, and qRT-PCR

| **Experiment** | **Gene ID** | **Gene** | **Primer names and sequences (5′-3′)** | **Product length (bp)** | |
| --- | --- | --- | --- | --- | --- |
| qPCR | 111036118 | MpGABR (GABAA receptor) | F: TCGTCAACGAGAAACAGTCG | | 229 |
|  |  |  | R: GAGTTTGGTCCCTTGTTCCA | |  |
|  | 111041856 | MpGABRAP (GABAA receptor-associated protein) | F: CTGGTACCCTCCGACCTG | | 202 |
|  |  |  | R: GTCCATAGACATTTTCAT | |  |
|  | 111036117 | MpGABRB (GABAA receptor subunit beta) | F: TGAAGTCGGTGTCACCATGT | | 232 |
|  |  |  | R: GGTGGTCGCTATGTGGAAGT | |  |
|  | 111028237 | maltase 2-like | F: TTTCGGCAGTGAAACTGAAA | | 164 |
|  |  |  | R: CAGATTGTGGTCGCAGTGTT | |  |
|  | 111028209 | uncharacterized protein | F: CCAGCATATGGCAAAGTCAA | | 183 |
|  |  |  | R: GGATGGACTGCTTGTTGGAT | |  |
|  | 111031319 | putative nuclease HARBI1 | F: TTAAAGGCTCGGTTCAGGTG | | 209 |
|  |  |  | R: CTTCCTCGAATGCCATCTTC | |  |
|  | 111035004 | AGLY_00338 | F: AAGGGCCAGCTTATCTCCAT | | 190 |
|  |  |  | R: GCCGTTTCATGTGGATTTTC | |  |
|  | 111036236 | troponin C-like | F: ACAACAACATGTCCGACGAA | | 168 |
|  |  |  | R: TTCAAAAAGTGCTGCGTTTG | |  |
|  | 111038117 | zinc finger MYM-type protein 1-like | F: TCATGAGCATTGAGCACCTC | | 215 |
|  |  |  | R: TAGCCGCACCACTAAGGACT | |  |
|  | 111037485 | uncharacterized protein | F: GCCAATCGCAAAAAGCTTAG | | 242 |
|  |  |  | R: AGCTGGCCCAGTTCAACTTA | |  |
|  | 111032528 | histone H3.2 | F: ACAGACCTGCGTTTCCAAAG | | 165 |
|  |  |  | R: ACGTTCTCCACGGATACGAC | |  |
|  | 111035341 | extensin-like isoform X1 | F: TACTCGTCACCGCACTTCAG | | 246 |
|  |  |  | R: CGATCGGTGGCGTTAGTAAT | |  |
|  | 100161709 | venom serine carboxypeptidase-like | F: AAACGGAAAGCACAAAATCG | | 225 |
|  |  |  | R: GGTTTTTCCACGAACTGCAT | |  |
|  | 111033337 | zinc finger and BTB domain-containing protein 24-like | F: TCCGTTGAAGGGTAAAGTCG | | 200 |
|  |  |  | R: AAGGTTGTCTCTCCGTGTGAA | |  |
|  | 111027646 | Actin binding protein 1 | F: AATTGGACGCGAACAAAAAC | | 153 |
|  |  |  | R: TTTCTTCGATTCCCCCTTCT | |  |
|  | 111032238 | 40S ribosomal protein S18 (PRS18) | F: CCTGTCCACCTTGTAGCGTT | | 132 |
|  |  |  | R: TGTGCTGGAACTTCTCAGGG  TGTGCTGGAACTTCTCAGGG  TGTGCTGGAACTTCTCAGGG | |  |
| RNAi | 111036118 | MpGABR | F: taatacgactcactatagggCTTGACGACGGGCAACTATT | | 325 |
|  |  |  | R: taatacgactcactatagggAATAGTTGCCCGTCGTCAAG | |  |
|  | 111041856 | MpGABRAP | F: taatacgactcactatagggTGAAGTTCCAGTACAAAG | | 183 |
|  |  |  | R: taatacgactcactatagggCTTTTTCTTGTCGAGCTCGC | |  |
|  | 111036117 | MpGABRB | F: taatacgactcactatagggCGAATTCATCCGGATACACC | | 250 |
|  |  |  | R: taatacgactcactatagggAGGGACACTTCGTTGGACAC | |  |
|  | ACY56286 | GFP | F: taatacgactcactatagggTCGGCGGCAATCCTGATCAA | | 476 |
|  |  |  | R: taatacgactcactatagggTCACAGGGTAAAATTCAGCA | |  |
| cloning | 111036118 | MpGABR | F: ATGACGTGTGGCGGCCGGCGG | | 2082 |
|  |  |  | R: TCAGTCTGCGCCCAGCAGCAC | |  |
|  | 111041856 | MpGABRAP | F: ATGAAGTTCCAGTACAAAGAA | | 354 |
|  |  |  | R: TCACACTCGTCCATAGACATTTTC | |  |
|  | 111036117 | MpGABRB | F: ATGACCGGCCGCGCCGCGCAC | | 750 |
|  |  |  | R: TCATACTTGCGTTGGAGATATTTT | |  |
| heterologous expression | 111036118 | MpGABR | F: CGCGGATCCGCGATGACGTGTGGCGGCCGGCGG | | 2105 |
|  |  |  | R: TCCCCCGGGGGTCAGTCTGCGCCCAGCAGCAC | |  |

| **Table S2.** Oligonucleotide sequences for PCR, the sgRNA synthesis template and the fragment of donor DNA for homology-directed repair (HDR) | |
| --- | --- |
| **Sequence name** | **Sequences (5’ to 3’)** |
| sgRNA-F (R122T) | TAATACGACTCACTATATTAGCGTATAGAAAACGACCT |
| sgRNA-R (R122T) | TTCTAGCTCTAAAACAGGTCGTTTTCTATACGCTAA |
| donor DNA (R122T) | GGACTTCACATTGGATTTTTACTTTCGTCAATTTTGGACCGATCCTCGTTTAGCGTATACAAAACGACCTGGTGTAGAAACACTATCGGTTGGATCAGAGTTCATTAAGAATATTTGGGTACCTGACACCTTTTTTGTAAATGAAAAACAATCATATTTTCACATTGCAACAACCAGTAATGAATTCATACGTGTGCATCATTCTGGATCGATAACAAGAAGTATTAG |
| Check-F | TACGTAAGTTCTCACTGCCAA |
| Check-R | GTTTGGATGTAGGGTTAGTTG |
| Note: The GenBank number of target sequence is KF881792.1 (*Drosophila melanogaster*). | |
